# Volumetric absorptive microsampling for lumateperone analysis: method validation and stability evaluation

**DOI:** 10.1007/s00216-025-06169-4

**Published:** 2025-11-19

**Authors:** Elisa Milandri, Roberta Di Lecce, Chiara Pia Iattoni, Andrea Armirotti, Tomaž Vovk, Roberto Mandrioli, Michele Protti, Laura Mercolini

**Affiliations:** 1 Research Group of Pharmaco-Toxicological Analysis (PTA Lab), Department of Pharmacy and Biotechnology (FaBiT), Alma Mater Studiorum - University of Bologna, Via Belmeloro 6, 40126 Bologna, Italy; 2 Department of Pharmacy and Biotechnology (FaBiT), Alma Mater Studiorum - University of Bologna, Corso d’Augusto 237, 47921 Rimini, Italy; 3 Analytical Chemistry Facility, Istituto Italiano Di Tecnologia, Via Morego 30, 16163 Genoa, Italy; 4 Department of Biopharmaceutics and Pharmacokinetics, University of Ljubljana, Aškerčeva Cesta 7, 1000 Ljubljana, Slovenia

**Keywords:** Lumateperone, Microsampling, Volumetric absorptive microsampling (VAMS), Solid-phase extraction (SPE), HPLC–MS/MS, Method development and validation

## Abstract

Lumateperone is a recently approved atypical antipsychotic for the treatment of schizophrenia, with moderate adverse effects. However, the risk of toxicity, mainly due to possible multidrug coadministration, makes the therapeutic drug monitoring (TDM) approach crucial for developing personalised therapies and reducing side effects. In the TDM research field, microsampling represents an innovative and effective approach based on the collection and analysis of small sample volumes (< 100 μL), exhibiting many advantages (such as enhanced analyte stability, minimally invasive procedures and simplified sample storage and transport) and improving patient adherence. For the first time, an analytical methodology based on microsampling was developed and fully validated for the analysis of lumateperone in capillary blood microsamples, by means of volumetric absorptive microsampling (VAMS) in comparison with plasma analysis, with the future perspective of supporting the TDM of psychiatric patients. A reliable and streamlined VAMS protocol was optimised, and an original HPLC–MS/MS method was developed and validated, granting satisfactory results on simulated samples in terms of linearity, precision, extraction yield and matrix effect. Method reliability was successfully tested by comparing the VAMS performance with standard in-tube fluid plasma, and short- and medium-term comparative stability assays confirmed the enhancement of analyte stability obtained in dried microsamples with respect to plasma samples. This is the first analytical method based on microsampling able to provide reliable data when compared to plasma analysis and promising for future TDM applications in patients treated with lumateperone, thus offering significant advantages over conventional blood/plasma sampling in terms of collection, storage and processing.

**Graphical abstract:**

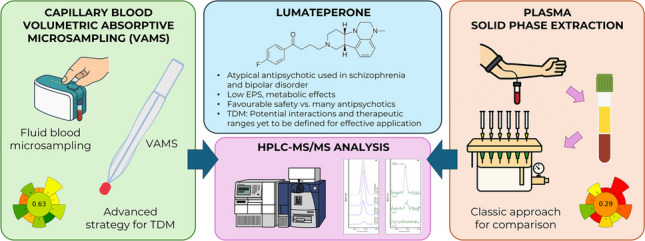

**Supplementary Information:**

The online version contains supplementary material available at 10.1007/s00216-025-06169-4.

## Introduction

Over the past several years, intensive pharmacological research in psychiatric disorders has led to the development of second-generation (atypical) antipsychotics. Compared to first-generation antipsychotics (neuroleptics), these compounds show improved efficacy and tolerability, mainly due to reduced or partial affinity for dopamine D_2_ receptors and strong interaction with various serotonin (5-HT) receptor subtypes, which together improve quality of life in patients with schizophrenia [1, 2].

Among these agents, lumateperone (LUM, also known as ITI-007) was approved by the U.S. Food and Drug Administration (FDA) in 2019 for the treatment of schizophrenia in adults, at the oral dose of 42 mg once daily. In 2021, its indication was extended to depressive episodes associated with bipolar I or II disorder, either as monotherapy or in combination with lithium or valproate [3, 4].

**Fig. 1:**
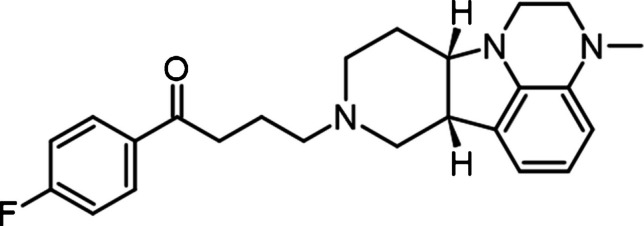
Chemical structure of lumateperone

The chemical structure of LUM (1-(4-fluorophenyl)−4-(3-methyl-2,3,6b,9,10,10a-hexahydro-1*H*-pyrido[3′,4′:4,5]pyrrolo[1,2,3-de]quinoxalin-8(7*H*)-yl)−1-butanone; Fig. 1) enables multimodal activity across dopaminergic, serotonergic, and glutamatergic neurotransmission [3–5]. It acts as an antagonist at 5-HT_2A_ receptors, a presynaptic partial agonist, and postsynaptic antagonist at dopamine D_2_ receptors, also modulating D_1_ and glutamate receptor pathways [6–10]. In addition, LUM inhibits the serotonin transporter (SERT), increasing serotonin availability [5, 10, 11]. Following administration, LUM reaches peak plasma levels within 1–2 h, with maximum concentrations observed after 3–4 h [12, 13]. It seems there are few active metabolites, such as IC200161, IC200565 (via CYP3A4-mediated dealkylation), and IC200231 (via ketone reduction) [9, 12, 14]. Despite its favourable safety profile, including lower risk of extrapyramidal and metabolic side effects [7, 9, 15], LUM metabolism via CYP3A4 and UGT raises concerns for drug-drug interactions, especially in polypharmacotherapy settings [6, 13, 16]. At present, no consolidated therapeutic plasma concentration ranges have been established for LUM. Nevertheless, validated analytical methods represent key enablers to generate the evidence base that will shape future therapeutic drug monitoring (TDM) recommendations. In fact, in this framework, TDM can be a valuable tool to personalise therapy, optimise efficacy, and minimise toxicity [17, 18]. However, conventional venous blood collection for TDM poses some crucial challenges: it requires relatively large sample volumes (1–5 mL), trained personnel, and cold-chain storage to avoid analyte degradation [19]. Moreover, psychiatric patients may experience discomfort or anxiety during venipuncture as an invasive procedure, creating additional barriers to TDM. Dried microsampling offers an attractive alternative. After fingerprick, once dried, these microsamples are stable at room temperature, facilitating easier storage and shipment [20, 21]. Among available technologies, volumetric absorptive microsampling (VAMS) allows collection of a fixed volume of blood (10–30 μL) using a porous polymeric tip, in an accurate way, regardless of fluid viscosity [21–23]. This mitigates key limitations of traditional dried blood spot (DBS) sampling, particularly haematocrit-related bias.

This study aimed to develop and validate a miniaturised bioanalytical method based on VAMS coupled to high-performance liquid chromatography–tandem mass spectrometry (HPLC–MS/MS) for the analysis of LUM in simulated capillary whole blood. Several research and review papers describe microsampling technologies for TDM of patients treated with CNS drugs, covering classic DBS, VAMS, capillary- and microfluidic-generated volumetric microsamples [19–28], thus underlining the growing importance of this field and the proven reliability of microsampling in the framework of TDM. However, very few studies are present in the scientific literature that specifically address LUM. Existing work mainly focuses on pharmacokinetics or formulation analysis [14, 29, 30] and no validated microsampling methods have been reported for TDM applications. The proposed methodology fills this gap, providing for the very first time a reliable alternative for LUM haematic analysis by using microsampling, also providing a comparison data set obtained from an originally developed extraction procedure for classic plasma analysis.

## Experimental

### Chemicals and solutions

All reagents were of analytical grade or better. LUM and LUM-D4 powders (≥ 98%) were purchased from Toronto Research Chemicals (Vaughan, ON, Canada), with LUM-D4 used as the internal standard (IS). HPLC-grade acetonitrile (ACN, > 99.9%), HPLC-grade methanol (MeOH, > 99.9%), and 95% formic acid (FA) were purchased from Sigma-Aldrich (Merck Life Science, Darmstadt, Germany). Ultrapure water (18.2 MΩ cm) was obtained using a Milli-Q apparatus from Millipore (Milford, MA, USA). LUM and IS stock solutions (1 mg/mL) were prepared by dissolving suitable amounts of pure powders in MeOH and kept at − 20 °C. Working solutions were prepared daily by dilution with a 30:70 (V/V) mixture of 0.1% FA in water and 0.1% FA in ACN. All solutions were prepared and stored in amber glass vials from Phenomenex (Torrance, CA, USA).

### Chromatography and mass spectrometry

The chromatographic system consisted of a Waters Alliance e2695 separation module coupled to a Waters Micromass Quattro Micro triple quadrupole mass spectrometry system (Milford, MA, USA). Separations were obtained on a Waters (Milford, MA, USA) Cortecs C18 column (100 mm × 2.1 mm i.d., 2.7 µm), kept at room temperature (RT) and equipped with a VanGuard Cortecs C18 guard column (50 × 2.1 mm i.d., 2.7 µm). The mobile phase consisted of a mixture of 0.1% (V/V) FA in water (solvent A) and 0.1% (V/V) FA in ACN (solvent B); gradient composition was as follows: 0.0–1.0 min, constant 30% B; 1.1–4.0 min, linear 30–85% B gradient; 4.1–5.0 min, constant 85% B; 5.1–5.5 min, linear 85–30% B gradient; 5.6–7.5 min, constant 30% B for column re-equilibration. Flow rate was set at 300 µL/min and 10-μL injections were carried out. Tandem mass spectrometry acquisition was carried out in multiple reaction monitoring (MRM) scan mode, through an electrospray ionisation (ESI) source operating in positive mode. MS working conditions were as follows: ion source voltage, 3.5 kV; ion source temperature, 120 °C; desolvation temperature, 350 °C; desolvation gas flow, 600 L/h; dwell times per channel, 300 ms; cone gas flow, 50 L/h. Nitrogen was used as desolvation gas and was generated from pressurised air by an N2 nitrogen generator (Claind, Lenno, Italy); collision gas was 99.995% argon (Fluido Tecnica, Firenze, Italy). MRM transitions were monitored as follows: [M + H]⁺ parent ions were m/z 394.23 for LUM and m/z 398.22 for LUM-D4, while selected product ions were m/z 165.1 and 169.1 for LUM and LUM-D4, respectively. Data acquisition and quantitative processing were accomplished using Waters MassLynx software, version 4.1.

### Sample collection and pretreatment

**Fig. 2:**
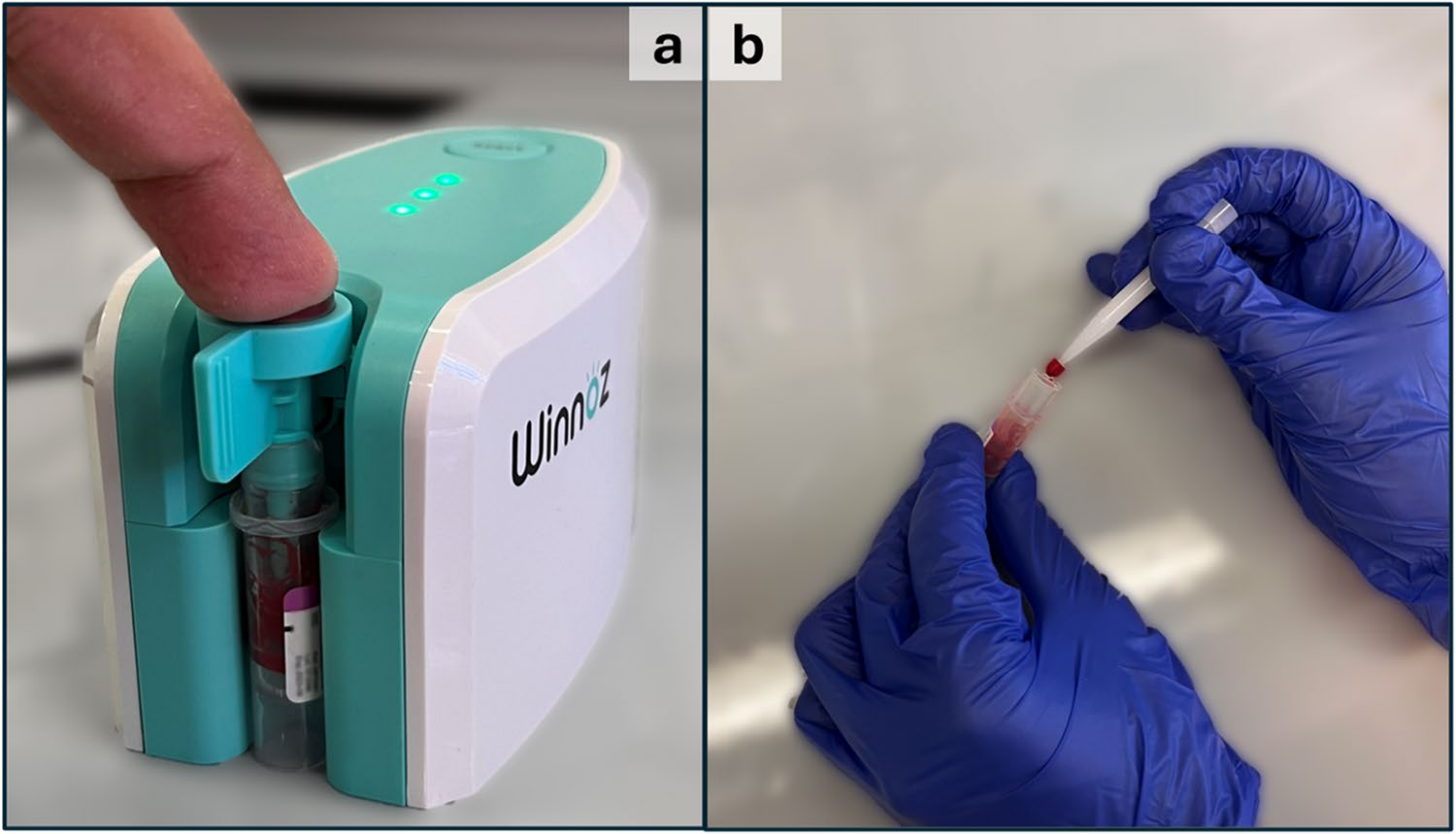
Blood collection for microsampling: (**a**) capillary blood collection under vacuum after fingerprick, (**b**) VAMS sampling

Capillary whole blood for method development and validation was collected from healthy volunteers using the Haiim vacuum device by Winnoz (New Taipei City, Taiwan). Following a fingerprick performed with a disposable lancet, the fingertip was positioned over the device’s designated inlet. Upon activation, the device aspirates up to 500 µL of blood through the inlet by vacuum action into an anticoagulant-coated reservoir (Fig. 2a). 20 μL VAMS devices, commercialised under the Mitra® trade name, were purchased from Neoteryx Microsampling Solutions by Trajan Scientific and Medical (Ringwood, Victoria, Australia). Sampling was performed by placing the VAMS device tip in contact with fluid capillary blood for 4 s, allowing uniform absorption and full tip saturation (Fig. 2b). Blood VAMS tips were fortified with 20 µL of standard mixtures containing LUM and IS at known concentrations. To obtain fortified samples, three different procedures potentially suitable for blood microsampling were investigated. In the first approach, the tip of a VAMS device (not yet used to sample blood) was put in contact for 4 s with the surface of a standard mixture containing LUM and IS, then left to dry for 45 min, used to collect blood (as described above), then dried for an additional 45 min. In the second approach, 20 µL of the standard mixture containing LUM and IS were directly pipetted onto the tip of a VAMS device (not yet used to sample blood), which was then dried for 45 min prior to blood sampling and drying for 45 min. In the last (third) approach, 20 µL of blood were collected using a VAMS device and allowed to dry for 45 min; the dried VAMS tip was then brought into contact with the surface of a standard mixture containing LUM and IS, then left to dry again for 45 min. The analyte and IS were extracted from all three types of dried VAMS tips using ultrasound-assisted extraction (UAE) by using 200 µL of MeOH for 15 min at an ultrasound power of 150 W. The resulting supernatant was brought to dryness exploiting a Thermo Fisher Sci. (Waltham, MA, US) Savant SpeedVac SPD 1030 vacuum concentrator and redissolved with 100 µL of a 30:70 (V/V) mixture of 0.1% FA in water and 0.1% FA in ACN prior to HPLC–MS/MS analysis. Fluid plasma was obtained from fluid whole blood by centrifugation. The plasma was then transferred to polypropylene tubes and stored at − 20 °C until sample pretreatment. An original solid phase extraction (SPE) protocol was developed, validated, and employed as the reference plasma standard procedure for comparison with whole blood VAMS results, using Agilent (Santa Clara, CA, US) BondElut C18 cartridges (50 mg, 1 mL) as follows: activation and conditioning with 3 × 1 mL MeOH, 3 × 1 mL H_2_O; sample loading with 250 μL plasma, 500 μL H_2_O, and 50 μL IS solution; washing with 2 × 1 mL H_2_O, 1 mL H_2_O/MeOH mixture (90:10, V/V), and 50 μL MeOH; elution with 1 mL MeOH. The extract was dried under vacuum and, finally, reconstituted in 100 μL of a 30:70 (V/V) mixture of 0.1% FA in water and 0.1% FA in ACN before injection into the chromatographic system.

### Method validation

Method validation was carried out according to current international guidelines for bioanalytical methods as reported by the International Council for Harmonisation of Technical Requirements for Pharmaceuticals for Human Use (ICH) [31] and the US Food and Drug Administration (FDA) [32].

#### Linearity

Linearity was assessed by fortifying blank blood VAMS obtained from healthy volunteers with 20 μL aliquots of standard mixtures containing LUM at seven different concentrations and IS at a constant concentration. The resulting fortified VAMS samples were subjected to the pretreatment procedure described above and injected into the HPLC–MS/MS system. The procedure was carried out in triplicate for each concentration. The analyte/IS peak area ratios obtained were plotted against the corresponding nominal LUM concentrations, and the calibration curves were set up using the linear least-square regression method. For sensitivity, the limit of quantitation (LOQ) and limit of detection (LOD) were calculated by examining 6 different fortified blood VAMS HPLC–MS/MS chromatograms and expressed as the analyte concentrations giving rise to peaks whose areas are 10 and 3 times the baseline noise, respectively.

#### Extraction yield and precision

Extraction yield and precision were evaluated by analysing blank blood VAMS samples obtained from healthy volunteers and fortified at three LUM concentrations representative of the calibration curve (and IS at a constant concentration) after subjecting them to the optimised sample pretreatment protocol described above. Analyte peak areas were compared to those obtained by injecting standard solutions at the same theoretical concentrations, and percentage extraction yields were calculated.

The same procedure was repeated six times within the same day in order to evaluate intraday precision and six times over six days to obtain interday precision, both expressed as percent relative standard deviation (RSD%).

#### Selectivity

Selectivity towards endogenous compounds was assessed by analysing blank VAMS samples collected from six healthy volunteers. The method can be considered selective if no endogenous peaks exceeding the analyte response at the LOD were observed at the analyte retention time.

#### Matrix effect and carryover

The matrix effect was evaluated by fortifying pretreated blank matrix extracts (i.e., obtained from blank VAMS samples processed according to the extraction protocol) with known amounts of the analyte at three different concentrations, corresponding to the lower, middle, and upper levels of the calibration range. The resulting peak areas were compared to those of standard solutions at equivalent concentrations, and the percentage relative error (RE%) was calculated.

Carryover was assessed by injecting a solvent blank immediately after a VAMS extract fortified at the upper limit of the linearity range. The absence of carryover was confirmed when no analyte signal exceeding 20% of the lower limit of quantification (LLOQ) and no IS signal above 5% was detected.

#### Stability

The stability of LUM in the dried blood matrix was assessed through short- and mid-term experiments using fortified blank blood VAMS samples (n = 3). Samples were spiked at two concentration levels (low and high) within the validated calibration range and stored at RT in sealable polyethylene bags containing desiccant, protected from light, humidity, and heat. Analyses were performed by HPLC–MS/MS at predefined time points (day 0, 1, 7, 14, 21, and 30). Short-term stability was considered acceptable if the mean analyte concentration remained within ± 20% of the initial value (i.e., recovery ≥ 80%). To simulate potential degradation scenarios, additional VAMS samples were stored under stress conditions (35 °C, uncontrolled humidity, and exposure to artificial light) and analysed at the same time intervals. For comparative purposes, plasma samples stored at −20 °C and −80 °C were also analysed after 0, 28, 56, and 90 days using the validated SPE-HPLC–MS/MS method developed and validated on purpose within this study to evaluate the medium-term stability of LUM in VAMS samples stored at RT vs. fluid matrix under standard biobank conditions.

## Results and discussion

### Development of sampling and pretreatment procedures

The sampling and pretreatment procedures using VAMS devices were fully developed and validated, assessing several key parameters. The initial focus was placed on confirming the accuracy of sample volume, and VAMS devices achieved comparable sampling accuracy to volumetric pipetting, showing no statistically significant differences in collected volumes [33]. Moreover, a contact time of 4 s is sufficient to fully saturate the 20-μL VAMS tips, and the intensity of the red colour from the absorbed whole blood provides a visual indication of complete tip filling. Three different procedures for preparing fortified VAMS samples were evaluated, varying the sequence of blood sampling and standard mixture addition. Regardless of the approach (application of the standard mixture by contact or pipetting before blood sampling, or post-sampling fortification), the results were comparable in terms of extraction yield (mean values ranging from 89.5% to 91.1%), precision (RSD < 0.7%), and matrix effect (5.8–6.5%). No significant differences were observed among the three protocols, confirming their practical interchangeability. Any of the tested workflows can thus be reliably adopted according to specific operational needs or logistical considerations. Subsequently, the pretreatment parameters were optimised in terms of the extraction yield of LUM and the IS from VAMS samples, including the extraction solvent, solvent volume, extraction time, and ultrasound-assisted extraction (UAE) conditions. A range of solvents (MeOH, ACN and water) and their mixtures of them were tested in different volume ratios (10:90, 30:70, and 50:50, V/V). Extraction volumes from 50 to 1000 μL, durations between 5 and 30 min, and ultrasound power levels from 50 to 200 W were assessed. The best extraction yield was achieved by UAE for 15 min at 150 W in 200 μL of MeOH. Extraction yields increased with solvent volume up to 200 μL, but higher volumes did not provide further improvement and instead increased matrix effect. Specifically, the mean matrix effect was 8.6% with 200 μL of MeOH, 9.6% with 300 μL, and 10.5% with 500 μL. A similar pattern was observed with extraction time, where longer durations did not significantly enhance extraction but led to more pronounced matrix effects. The combination of VAMS and UAE significantly reduced both solvent consumption and extraction time, while also simplifying the handling of biological samples compared to conventional liquid-based methods. The optimised parameters are summarised in Table 1.

**Table 1:** Results of VAMS pretreatment optimisation for LUM analysis

Parameter	Tested conditions	Optimal value	Extraction yield (%)	Matrix effect (RE%)	Notes
Solvent type	100% MeOH, ACN, H_2_O; MeOH/ACN, MeOH/H_2_O, ACN/H_2_O mixtures (10:90, 30:70, 50:50—V/V)	100% MeOH	> 88.0 (89.5 for IS)	< 6.9 (5.1 for IS)	Pure MeOH gave best yield
Solvent volume	50, 100, 200, 300, 500, 1000 μL	200 μL			Higher volumes increased matrix effect
Extraction time	5, 10, 15, 20, 30 min	15 min			Longer times increased matrix effect
Ultrasound power	50, 100, 150, 200 W	150 W			Higher power did not improve yield

In parallel, a dedicated SPE procedure was developed for plasma samples to serve as a reference method for comparison with the VAMS-based approach. The choice of C18 sorbent material for SPE was guided by the physicochemical properties of the target analyte and by the versatility of C18 phases towards non-polar to moderately polar compounds. Then, the extraction protocol was systematically optimised by fine-tuning the sample loading, washing, and elution conditions, aiming to maximise both analyte recovery and matrix clean-up efficiency. The final protocol consisted of cartridge activation with 3 × 1 mL MeOH and 3 × 1 mL water, followed by sample loading (250 µL plasma + 500 µL water + 50 µL IS standard solution). The washing step was performed sequentially with 2 × 1 mL water, 1 mL of 90:10 (V/V) water/MeOH, and 50 µL MeOH. Elution was carried out with 1 mL MeOH; the eluate was then dried under vacuum and reconstituted in 100 µL of a 30:70 (V/V) mixture of 0.1% FA in water and in ACN.

### HPLC–MS/MS conditions

To establish suitable MS/MS conditions, working solutions of LUM and LUM-D4 (1 μg/mL in 0.1% formic acid in ACN/water, 70:30, V/V) were directly infused into the ESI source at a flow rate of 10 μL/min using a syringe pump. Full-scan mass spectra were acquired in the positive ion mode over an m/z range of 100–600. The [M + H]⁺ ions were observed at m/z 394.23 for LUM and m/z 398.22 for LUM-D4. The most intense product ions were detected at m/z 165.1 and 169.1, respectively, and selected for MRM.

**Fig. 3:**
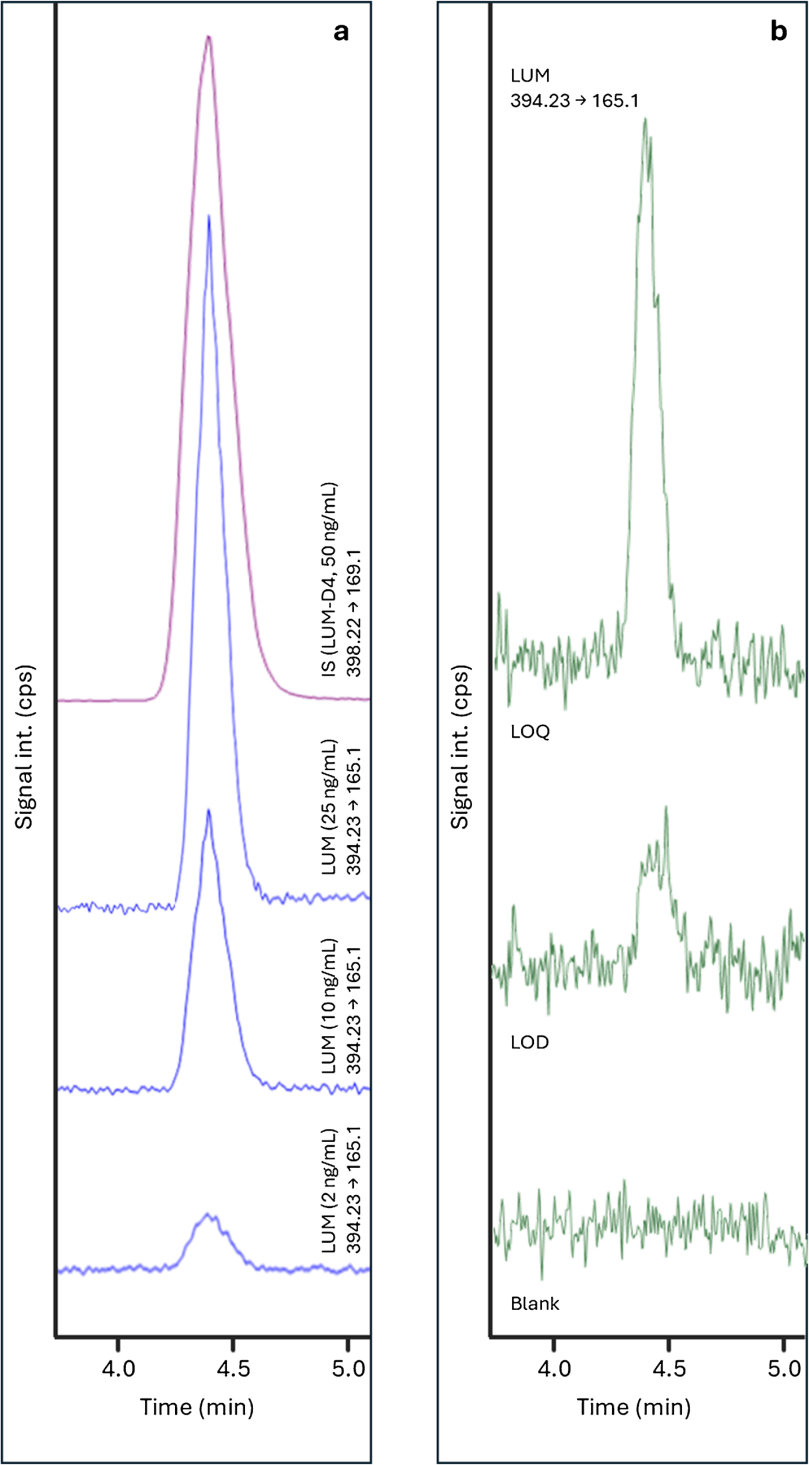
MRM HPLC–MS/MS chromatograms of: (a) four blank VAMS samples fortified with LUM at different concentrations and IS at 50 ng/mL, (b) one blank VAMS sample and two blank VAMS sample fortified with LUM at LOD and LOQ levels

For chromatographic method development, two stationary phases equipped with guard columns were tested: a C8 column (100 × 2.1 mm, 2.7 µm) and a C18 column (100 × 2.1 mm, 2.7 µm). In both cases, the mobile phase consisted of 0.1% formic acid in water (component A) and 0.1% formic acid in acetonitrile (component B). Gradient profiles and flow rates were systematically optimised for each configuration. The C18 column (Waters Cortecs C18, 100 × 2.1 mm, 2.7 µm) ultimately provided superior chromatographic performance in terms of peak shape, signal intensity, and overall sensitivity. The optimised elution program was: 0.0–1.0 min, 30% B; 1.1–4.0 min, linear increase to 85% B; 4.1–5.0 min, held at 85% B; 5.1–5.5 min, return to 30% B; 5.6–7.0 min, re-equilibration. LUM and the IS were eluted at 4.4 min, under a flow rate of 300 μL/min (Fig. 3).

### Method validation

The developed HPLC–MS/MS method for LUM analysis, coupled to the blood-VAMS approach, was systematically validated according to standard guidelines in terms of linearity, extraction yield, precision, selectivity, matrix effects, carryover, and stability.

To assess the reliability of the miniaturised method in comparison with a classic fluid analysis approach, conventional plasma analysis after SPE was also validated for the same parameters.

#### Linearity

To assess the linearity of the analytical method, blank spiked VAMS samples fortified with solutions of LUM at seven concentrations within the 0.5–250 ng/mL range were analysed. The calibration curve was obtained over the specific concentration range, with good linearity (*r*^2^ = 0.9992). LOD and LOQ were found to be 0.2 ng/mL and 0.5 ng/mL respectively, highlighting a satisfactory method sensitivity.

Linearity was also tested in blank spiked plasma samples from healthy volunteers after SPE.

Detailed linearity data are presented in Table 2.

**Table 2:** Summary of method validation results for LUM analysis in fortified blood VAMS and fluid plasma samples

Linearity											
		Calibration range (ng/mL)		*r*^*2*^				LOD (ng/mL)		LOQ (ng/mL)	
Compound		Blood VAMS	Plasma SPE	Blood VAMS		Plasma SPE		Blood VAMS	Plasma SPE	Blood VAMS	Plasma SPE
LUM		0.5–250	0.3–250	0.9992		0.9989		0.2	0.1	0.5	0.3
Compound	Concentration	Extraction yield (%)^a^		Precision (%RSD)^b^				Matrix effect (%RE)^a^		Carryover (%)^a^	
				Intraday		Interday					
		Blood VAMS	Plasma SPE	Blood VAMS	Plasma SPE	Blood VAMS	Plasma SPE	Blood VAMS	Plasma SPE	Blood VAMS	Plasma SPE
LUM	Low	88.1	87.4	10.6	11.2	11.1	11.8	6.8	8.4	6.3	7.6
	Medium	91.3	90.8	9.7	10.5	10.1	11.1	6.2	7.9		
	High	93.7	92.1	8.9	9.3	9.5	9.7	5.6	7.1		
IS	Constant	89.5	90.2	7.4	8.7	7.9	9.3	5.1	6.5	2.1	2.9

^a^*n* = 3

^b^*n* = 6

#### Extraction yield and precision

Extraction yield and precision assays were assessed on blank VAMS from healthy volunteers fortified with mixtures of the analyte at three concentrations (corresponding to the lower limit, a middle point, and a high value of the calibration curve) and IS at 50 ng/mL (constant concentration).

Extraction yield and precision were also tested in blank plasma samples from volunteers after SPE.

Complete on extraction yield and precision results are provided in Table 2.

#### Selectivity

The developed method showed high selectivity for LUM analysis, with no interference observed from endogenous compounds in blank blood samples obtained from six different volunteers. Blank plasma samples obtained from the same subjects were also successfully tested.

#### Matrix effect and carryover

Matrix effect was consistently low, with RE values ≤ 6.9% indicating minimal signal suppression or enhancement. This result suggested that the presence of blood/plasma components did not interfere with analyte identification and quantitation.

No significant carryover was observed: injection of VAMS blank samples (and plasma samples) right after the highest calibrators yielded signals lower than 6.4% of the LOQ level (< 2.2% for IS). Both matrix effect and carryover were low for VAMS and satisfactory for plasma, thus not affecting method reliability.

Complete matrix effect and carryover results are reported in Table 2.

#### Comparison with conventional plasma analysis

To assess the reliability of the miniaturised method, conventional plasma analysis was also carried out: the results obtained from VAMS were compared to those resulting from the analysis of fluid plasma using SPE. Plasma-SPE was chosen as the reference workflow because it represents the most widely established and validated approach in conventional, non-miniaturised bioanalysis. To the best of our knowledge, no LUM methods are currently available in the literature. For this reason, establishing a robust plasma SPE protocol was considered a necessary benchmark for comparison with the novel VAMS method. As shown in Table 2, both approaches provided comparable results, with VAMS samples yielding higher extraction efficiencies and lower matrix effects, with overall analytical performance in line with regulatory standards. These findings further support the suitability of the VAMS approach as a reliable and ready-to-implement strategy for LUM monitoring in dried blood microsamples. Beyond analytical performance, the environmental sustainability of the two workflows was also considered. To this end, the greenness of the VAMS-HPLC–MS/MS protocol and the plasma-SPE workflow was assessed using the AGREEprep metric [34]. As illustrated in the Supplementary Material (Fig. S1), the VAMS-based procedure achieved a higher greenness score than plasma-SPE (0.63 vs. 0.29), mainly due to its lower blood and solvent requirements, reduced consumable use, and simplified logistics. These findings underline the additional sustainability advantage of VAMS, complementing its analytical robustness and supporting its translational potential.

#### Stability

To evaluate the reliability over time of the proposed microsampling approach, LUM stability was assessed under both short- and mid-term conditions, also comparing VAMS to conventional plasma storage. Short-term stability was first investigated by comparing fortified blood VAMS samples stored for 30 days under controlled room conditions with those subjected to sub-optimal environmental stress. In the latter case, samples were kept at 35 °C, exposed to artificial light, and under uncontrolled humidity. As expected, these stress conditions led to a moderate decrease in analyte stability compared to RT storage. Nonetheless, LUM recoveries remained within the acceptable limits defined by current bioanalytical guidelines, with average extraction not lower than 87.9 ± 6.8% (Table 3), thus confirming the good resilience of VAMS samples even under unfavourable storage conditions.

**Table 3:** Stability of LUM in VAMS and plasma under different storage conditions

Short-term stability – Ideal vs. sub-optimal VAMS storage						
Time points (days)	0	1	7	14	21	30
LUM signal (%)^a^						
VAMS (ideal)	100	98.2 ± 5.2	97.5 ± 5.5	96.4 ± 5.7	95.6 ± 6.3	94.7 ± 6.5
VAMS (sub-optimal)	100	95.1 ± 5.9	92.8 ± 6.1	89.4 ± 6.3	88.1 ± 6.6	87.9 ± 6.8
Medium-term stability – Blood VAMS (RT) vs. fluid plasma (-20°C and -80°C)						
Time points (days)	0		28	56		90
LUM signal (%)^a^						
Blood VAMS (RT)	100		94.9 ± 5.3	93.9 ± 5.6		93.0 ± 6.0
Fluid plasma (-20°C)	100		92.4 ± 5.7	90.5 ± 6.4		88.4 ± 6.9
Fluid plasma (-80°C)	100		93.6 ± 5.5	91.6 ± 5.8		90.2 ± 6.6

^a^*n* = 3

Mid-term stability was then evaluated by storing VAMS samples at RT for up to 90 days and comparing them to matched plasma samples stored at −20 °C and −80 °C. Across all timepoints, LUM proved stable in both matrices, with a mean extraction of 93.0 ± 6.0% in VAMS, 88.4 ± 6.9% in plasma at −20 °C, and 90.2 ± 6.6% in plasma at −80 °C, after 90 days (Table 3). Notably, VAMS samples showed slightly better stability despite being stored at ambient conditions, further supporting the reliability of these dried matrices. Overall, these findings confirm that the dried nature of VAMS contributes to improved analyte stability, supporting its applicability in real-world settings such as home sampling and room temperature transport and storage.

## Conclusion

To the best of our knowledge, this is the first study presenting a method for LUM quantitation in both whole blood and plasma, respectively proposing a microsampling-based approach and a classic SPE sample treatment. A novel analytical method based on VAMS and HPLC–MS/MS was developed and fully validated for LUM haematic quantitation in simulated samples. The method showed satisfactory results during validation on blank spiked samples, and good reliability when blood data are compared to those obtained from the protocol developed in parallel for classic fluid plasma treated by SPE.

One of the main potential issues related to dried blood microsamples concerns the stability of the analytes over time, thus requiring careful control of collection, storage, and transport conditions, as well as of the analytical processing step itself. A short- and medium-term stability study was therefore conducted herein, comparing the reliability of VAMS samples stored under ideal and sub-optimal ambient conditions with that of liquid plasma samples stored in freezers under controlled temperatures. The conclusion reached was that LUM stability in VAMS is superior under the tested conditions.

This original method therefore enables the analytical processing of both dried blood microsamples and liquid plasma samples, offering a highly reliable approach with promising future applications for TDM of patients treated with LUM. Given the scarce data currently available in the literature on this drug, the present study provides a validated analytical workflow that can enable future TDM applications of LUM, representing a significant advancement in its analytical management.

## Supplementary Information

Below is the link to the electronic supplementary material.

Supplementary Material 1 (DOC 151 KB)

## Data Availability

The authors declare that the data supporting the findings of this study are available within the paper. Should any raw data files be needed in another format, they are available from the corresponding author upon reasonable request.
