# Supplementary material for: Volumetric absorptive microsampling for lumateperone analysis: method validation and stability evaluation
Source: Anal Bioanal Chem. 2025 Nov 19;418(2):643–53. doi: 10.1007/s00216-025-06169-4 (PMC12783156; doi:10.1007/s00216-025-06169-4)
Supplement: Supplementary file 1 — Supplementary Material 1 (DOC 151 KB) [file 216_2025_6169_MOESM1_ESM.doc]

**Volumetric absorptive microsampling for lumateperone analysis: Method validation and stability evaluation**

Elisa Milandri^1#^, Roberta Di Lecce^2#^, Chiara Pia Iattoni^1^, Andrea Armirotti^3^, Tomaž Vovk^4^, Roberto Mandrioli^2^, Michele Protti^1^*, Laura Mercolini^1^

^1^ Research group of Pharmaco-Toxicological Analysis (PTA Lab), Department of Pharmacy and Biotechnology (FaBiT), Alma Mater Studiorum - University of Bologna, Via Belmeloro 6, 40126 Bologna, Italy

^2^ Department for Life Quality Studies (QuVi), Alma Mater Studiorum - University of Bologna, Corso d’Augusto 237, 47921 Rimini, Italy

^3^ Analytical Chemistry Facility, Istituto Italiano di Tecnologia, Via Morego 30, 16163 Genova, Italy

^4^ Department of Biopharmaceutics and Pharmacokinetics, University of Ljubljana, Aškerčeva cesta 7, 1000 Ljubljana, Slovenia

# These authors contributed equally to this work.

* Corresponding Author:

Michele Protti - Email: michele.protti2@unibo.it

Contributing authors: elisa.milandri3@unibo.it; roberta.dilecce2@unibo.it; chiarapia.iattoni@studio.unibo.it; andrea.armirotti@iit.it; tomaz.vovk@ffa.uni-lj.si; roberto.mandrioli@unibo.it; michele.protti2@unibo.it; laura.mercolini@unibo.it.

**SUPPLEMENTARY MATERIAL**

**AGREEprep evaluation of the greenness of the proposed sample preparation workflows: VAMS-HPLC-MS/MS vs. SPE-HPLC-MS/MS**

The greenness of the two sample preparation workflows developed in this study (VAMS–HPLC-MS/MS and plasma–SPE–HPLC-MS/MS) was assessed using the Analytical Greenness Metric for Sample Preparation (AGREEprep) [Wojnowski W, Tobiszewski M, Pena-Pereira F, Psillakis E, AGREEprep - Analytical Greenness Metric for Sample Preparation, TrAC Trends Anal. Chem. 2022; 149: 116553. https://doi.org/10.1016/j.trac.2022.116553].
AGREEprep evaluates sample preparation procedures according to ten criteria derived from the principles of green sample preparation. Each criterion can be weighted according to its relevance, and the results are expressed both as a global score (0–1 scale) and as a color-coded radar chart, where green indicates more sustainable practices and yellow-orange-red indicate progressively lower performance (Figure S1):

- The VAMS workflow achieved a higher AGREEprep score, mainly due to its reduced blood volume requirements, lower solvent consumption, and simplified logistics for sample storage and transport.
- The plasma–SPE workflow received a lower score, reflecting its larger sample and solvent needs and more complex handling.

**VAMS**

**SPE**

**Supplementary Figure S1.** AGREEprep pictograms obtained for the

VAMS-HPLC-MS/MS (left) and SPE-HPLC-MS/MS (right) workflows for LUM analysis. The higher score achieved by the VAMS protocol mainly reflects its reduced sample and solvent requirements and simplified logistics, underlining its sustainability advantage over conventional plasma-SPE.
